# Supplementary material for: Bayesian refinement of protein structures and ensembles against SAXS data using molecular dynamics
Source: PLoS Comput Biol. 2017 Oct 18;13(10):e1005800. doi: 10.1371/journal.pcbi.1005800 (PMC5662244; doi:10.1371/journal.pcbi.1005800)
Supplement: S1 Archive — (BZ2) [file pcbi.1005800.s011.tar.bz2 › gromacs_4.62_bayesian_saxs_refi/share/html/online/g_nmtraj.html]

g\_nmtraj


| g\_nmtraj Main Table of Contents |

**VERSION 4.6  
Sat 19 Jan 2013**

---

### Description

g\_nmtraj generates an virtual trajectory from an eigenvector,
corresponding to a harmonic Cartesian oscillation around the average
structure. The eigenvectors should normally be mass-weighted, but you can
use non-weighted eigenvectors to generate orthogonal motions.
The output frames are written as a trajectory file covering an entire period, and
the first frame is the average structure. If you write the trajectory in (or convert to)
PDB format you can view it directly in PyMol and also render a photorealistic movie.
Motion amplitudes are calculated from the eigenvalues and a preset temperature,
assuming equipartition of the energy over all modes. To make the motion clearly visible
in PyMol you might want to amplify it by setting an unrealistically high temperature.
However, be aware that both the linear Cartesian displacements and mass weighting will
lead to serious structure deformation for high amplitudes - this is is simply a limitation
of the Cartesian normal mode model. By default the selected eigenvector is set to 7, since
the first six normal modes are the translational and rotational degrees of freedom.

### Files

| option | filename | type | description |
| --- | --- | --- | --- |
| **-s** | topol.tpr | Input | Structure+mass(db): tpr tpb tpa gro g96 pdb |
| **-v** | eigenvec.trr | Input | Full precision trajectory: trr trj cpt |
| **-o** | nmtraj.xtc | Output | Trajectory: xtc trr trj gro g96 pdb |

### Other options

| option | type | default | description |
| --- | --- | --- | --- |
| **-[no]h** | bool | no | Print help info and quit || **-[no]version** | bool | no | Print version info and quit || **-nice** | int | 19 | Set the nicelevel || **-eignr** | string | 7 | String of eigenvectors to use (first is 1) || **-phases** | string | 0.0 | String of phases (default is 0.0) || **-temp** | real | 300 | Temperature (K) || **-amplitude** | real | 0.25 | Amplitude for modes with eigenvalue<=0 || **-nframes** | int | 30 | Number of frames to generate |

---

http://www.gromacs.org  
gromacs@gromacs.org
